# Dr. Walker's Case of Vacciolation

**Published:** 1804-11-01

**Authors:** John Walker

**Affiliations:** Salisbury Square


### *To the Editors of the Medical and Phyfical Journal.*

Gentlemen,

In the long paper I sent you a few days ago, I ventured to offer my doubts on the accuracy of the conclusions so positively formed by some medical men on the Case in Fulwood's Rents, (p. 384); and my scepticism is not lessened by the circumstances of a Case, which has occurred at the Central House within the last twelve days.

The history of it will need no comment On the 12th instant, an alarmed mother came to me with her infant. "Sir, I have left a child at home that has been ill for some days, and now the small-pox are coming out; what shall I do for this infant? they have slept together all along, and I am afraid it may already have it in its blood." Let me inoculate it, by all means; if the child be not already infected, the inoculation will prevent it; if infected, it will arrest the progress of the dreadful disease, and lessen its sufferings and the danger. Of the certainty of this I have continual experience; for when the small-pox breaks out in any corner of this metropolis, mothers from such neighbourhood, come flocking to me with their children; those not yet infected escape; those infected have it mildly, the vacciolous vesicle in such instances exhibiting its characteristic appearances without apparent diminution.

Yesterday the mother presented her infant with a complete areola on each arm; and was assured, that it was secured to her. The poor woman had piously hung over her other child till, dying in her arms, it had left on her afflicted countenance the marks of that disease which closed its eyes in death. On her grief and vigil-worn

 cheek cheek, to which she had fondly pressed her suffering child, two large variolous pustules presented their hideous figure. What a lamentable thing that the other child was not inoculated! "Sir, I was quite easy about its safety; for some time ago it had a very great eruption; and no less than four doctors assured me that it was the small-pox."

Address of the protected child.—Richard Thomas, No. 6, Dorrington Street, Brook's Market; aged one year, three months.

*Salisbury Square*, 23 *x*, 1804. JOHN WALKER.

